# Impact of established prognostic factors and molecular subtype in very young breast cancer patients: pooled analysis of four EORTC randomized controlled trials

**DOI:** 10.1186/bcr2908

**Published:** 2011-06-24

**Authors:** Jos A van der Hage, J Sven D Mieog, Cornelis JH van de Velde, Hein Putter, Harry Bartelink, Marc J van de Vijver

**Affiliations:** 1 Division of Surgical Oncology, The Netherlands Cancer Institute, Plesmanlaan 121, Amsterdam, 1066 CX, The Netherlands; 2 Department of Surgery, Leiden University Medical Center, Albinusdreef 2, Leiden, 2333 ZA, The Netherlands; 3 Department of Medical Statistics, Leiden University Medical Center, Albinusdreef 2, Leiden, 2333 ZA, The Netherlands; 4 Division of Radiation Oncology, The Netherlands Cancer Institute, Plesmanlaan 121, Amsterdam, 1066 CX, The Netherlands; 5 Department of Pathology, Academic Medical Center, Meibergdreef 9, Amsterdam, 1105 AZ, The Netherlands; 6 Division of Diagnostic Oncology, The Netherlands Cancer Institute, Plesmanlaan 121, Amsterdam, 1066 CX, The Netherlands

## Abstract

**Introduction:**

Young age at the time of diagnosis of breast cancer is an independent factor of poor prognosis. In many treatment guidelines, the recommendation is to treat young patients with adjuvant chemotherapy regardless of tumor characteristics. However, limited data on prognostic factors are available for young breast cancer patients. The purpose of this study was to determine the prognostic value of established clinical and pathological prognostic factors in young breast cancer patients.

**Methods:**

Data from four European Organisation for Research and Treatment of Cancer (EORTC) clinical trials were pooled, resulting in a dataset consisting of 9,938 early breast cancer patients with a median follow-up of 11 years. For 549 patients aged less than 40 years at the time of diagnosis, including 341 node negative patients who did not receive chemotherapy, paraffin tumor blocks were processed for immunohistochemistry using a tissue microarray. Cox proportional hazard analysis was applied to assess the association of clinical and pathological factors with overall and distant metastasis free survival.

**Results:**

For young patients, tumor size (*P *= 0.01), nodal status (*P *= 0.006) and molecular subtype (*P *= 0.02) were independent prognostic factors for overall survival. In the node negative subgroup, only molecular subtype was a prognostic factor for overall survival (*P *= 0.02). Young node negative patients bearing luminal A tumors had an overall survival rate of 94% at 10 years' follow-up compared to 72% for patients with basal-type tumors.

**Conclusions:**

Molecular subtype is a strong independent prognostic factor in breast cancer patients younger than 40 years of age. These data support the use of established prognostic factors as a diagnostic tool to assess disease outcome and to plan systemic treatment strategies in young breast cancer patients.

## Introduction

The incidence of early stage breast cancer in young women is increasing. At present, breast cancer at a young age, that is, less than 40 years, accounts for approximately 5 to 7.5% of the total number of cases diagnosed each year in Western Europe and the US [1-3]. Based upon multiple retrospective analyses that demonstrated the unfavorable impact of young age on prognosis in breast cancer, several current consensus guidelines have included age ≥ 35 years as an absolute indication for adjuvant systemic chemotherapy irrespective of other tumor characteristics [4-6]. These guidelines imply that for young patients with favorable tumor features such as small tumor size and negative axillary nodal status, adjuvant chemotherapy and hormonal therapy for patients with hormone receptor positive tumors is advised although absolute treatment benefits are not well known. Moreover, the long-term toxicity of adjuvant chemotherapy and the implications of possible fertility impairment and premature menopause are of particular concern in young women [7]. In addition to an increased risk of developing distant metastases, local recurrence rates after mastectomy or breast conserving therapy are also higher than in older patients [5]. It has been demonstrated that an additional boost dose of radiotherapy after breast-conserving surgery decreases the risk of local recurrence especially in young women [8], but these loco-regional recurrence rates are still significantly higher compared with mastectomy in the young patients.

Retrospective analyses have demonstrated breast cancer at a very young age to be associated with higher grade, estrogen receptor negative tumors, a more advanced stage of disease at the time of diagnosis, and the presence of BRCA-1 or -2 germline mutations [9-13]. Recent gene expression profiling studies showed that tumors of young women showed a higher probability of PI3K, Myc, and β-catenin deregulation and lower mRNA levels of estrogen receptor-α, estrogen receptor-β and progesterone receptor, but higher levels of HER2, and epidermal growth factor receptor [14,15].

More refined knowledge of prognostic factors in young breast cancer patients will be of use in guiding therapy, including adjuvant chemotherapy, in these women. The prognostic value of molecular subtype based on immunohistochemistry is uncertain within the group of young breast cancer patients. Therefore, we pooled the data of four randomized European Organisation for Research and Treatment of Cancer (EORTC) early stage breast cancer trials and collected and characterized tumor material of patients younger than 40 years in order to perform multivariate prognostic factor analyses.

## Materials and methods

The data used in this study were obtained from four randomized phase III EORTC clinical trials that included patients with early stage breast cancer. The trials randomized between two types of loco-regional therapy and between different timing of the same type of systemic therapy. The detailed features of these trials have been described in detail previously [16-19]. The trial protocols are summarized below:

EORTC trial 10801 (1980 to 1986, median follow-up 13.4 years) was conducted in order to assess the safety of breast conserving treatment. Patients were randomized between breast conserving surgery combined with radiotherapy and modified radical mastectomy. Six cycles of adjuvant chemotherapy with cyclophosphamide 100 mg/m^2 ^given orally on days 1 to 14, methotrexate 40 mg/m^2 ^given intravenously on Days 1 and 8, and 5-fluorouracil 600 mg/m^2 ^(CMF) given intravenously on Days 1 and 8, were indicated for all node-positive patients under the age of 55. No information was collected on hormonal therapy. A total number of 902 patients were randomized of which 113 patients aged less than 40 years at the time of diagnosis [16].

EORTC trial 10854 (1986 to 1991, median follow-up 10.8 years) studied the question of whether one course of peri-operative chemotherapy given directly after surgery yields better results in terms of treatment outcome than surgery alone. Peri-operative chemotherapy consisted of one single course of doxorubicin 50 mg/m^2^, 5-fluorouracil 600 mg/m^2^, and cyclophosphamide 600 mg/m^2 ^(FAC), administered intravenously within 36 hours after surgery. Node-positive premenopausal patients in the peri-operative chemotherapy group were recommended to receive an additional five cycles of CMF. Node-positive premenopausal patients in the surgery alone group were advised to receive one conventional course of FAC followed by five cycles of CMF. Prolonged adjuvant systemic treatment was left to the discretion of the local investigators. A total number of 2,795 patients were included of which 396 patients aged less than 40 years at time of diagnosis [17].

EORTC trial 10902 (1991 to 1999, median follow-up 10 years) was set up to compare pre-operative adjuvant chemotherapy with postoperative chemotherapy. Chemotherapy consisted of four cycles of 5-fluorouracil 600 mg/m^2^, epirubicin 60 mg/m^2^, and cyclophosphamide 600 mg/m^2 ^(FEC) administered intravenously, at three-weekly intervals. A total number of 698 patients were randomized of which 125 patients aged less than 40 years at time of diagnosis [18,20].

EORTC trial 22881 (1989 to 1996, median follow-up 10.8 years) studied the value of a boost dose after primary breast conserving surgery. Patients with stage I or II breast cancer who had undergone microscopically complete surgical removal of the tumor and axillary dissection were randomly assigned to undergo 50-Gy irradiation of the whole breast with or without an additional dose of 16 Gy to the tumor bed. Patients with a microscopically incomplete excision were assigned to receive booster doses of 10 or 26 Gy. Patients with axillary lymph node involvement received adjuvant systemic therapy: premenopausal patients received chemotherapy (CMF, FEC, or FAC), and postmenopausal patients received tamoxifen. A total number of 5,569 patients were randomized of which 558 patients aged less than 40 years at time of diagnosis [8,19].

Adjuvant hormonal therapy for premenopausal hormone receptor positive patients was not yet recommended at the time these trials were conducted. In the oldest two trials, tamoxifen administration was not even recorded. In the trials where tamoxifen use was recorded, less than 5% of patients ≥ 40 years received tamoxifen. Therefore, we assumed that only a very small fraction of the young patients in these studies received tamoxifen.

### Collection of tumor material and immunohistochemistry

A request was sent to participating institutions to submit paraffin blocks containing a representative part of the tumor from all patients aged less than 40 years at the time of diagnosis except for those who had participated in EORTC trial 10902 and received neoadjuvant chemotherapy (this group of patients was excluded for this study to avoid the influence of down-staging by preoperative chemotherapy). Tumor tissue was collected and processed for immunohistochemistry using a tissue microarray. Three core biopsies of 0.6 mm were taken from every tumor specimen and placed in a so-called donor block. This procedure has been described previously [21-24]. A representative standard histological section from each individual tumor was stained with H&E to assess tumor type, to perform histological grading according to Elston and Ellis, and to assess the presence and extent of lymphangio invasion (none versus one to five versus more than five vessels) [25]. ER, PgR, HER2 and P53 expression levels were assessed using immunohistochemistry. Detailed procedures have been described previously [26-28]. In summary, a tissue microarray slide was stained and scored counting the percentage of positive cells and taking the mean value of the three tumor biopsies. For estrogen receptor expression, tumors with > 1% of the tumor cells showing nuclear staining were considered positive. For progesterone receptor expression, tumors with > 10% of the tumor cells showing nuclear staining were considered positive. For P53 accumulation, a semi-quantitative system was used based on the sum of the mean staining intensity (0 to 3; none to strong) and an estimation of the percentage of positive cell nuclei (0 to 4; 0% to > 75%); this allowed a sum score of 0 to 7, with staining ≥ 4 being considered positive [26,27]. HER2 expression was scored estimating the level of membranous staining (0, 1+, 2+, or 3+). Strong membranous staining in > 30% of tumor cells (3+) was considered positive. The molecular breast cancer subtypes were approximated using histological grade and the ER, PgR, and HER2 status of the primary tumor. Patients were categorized as follows: luminal A (ER+ or PgR+ and HER-2- and grade 1 or 2), luminal B (ER+ or PgR+ and HER-2+; or ER+ or PgR+ and HER2- and grade 3), HER-2 (ER- and PgR- and HER-2+), and basal type (ER- and PgR- and HER-2-). Estimations of tumor grade and protein expression levels were scored by two investigators (MJV and JAH) simultaneously who had to come to an agreement in case of different views.

### Statistical analyses

Data analysis was performed at the Leiden University Medical Center using SPSS for Mac (version 18.0; SPSS, Chicago, IL, USA). The Chi-square test was used to compare the distribution of baseline characteristics among groups. Endpoints studied were overall survival and distant metastasis free survival. Overall survival time was defined as the time between randomization and death from any cause. Distant metastasis-free survival time was defined as time to distant metastasis or death if the latter event occurred before a distant metastasis was diagnosed. Survival analyses were performed using the Kaplan Meier method. Covariates included, patient age, and tumor- and treatment related characteristics. Tumor characteristics were tumor size, nodal status, histological grade, hormone receptor status, HER2 status, P53 status, molecular subtype and lymphangio invasion. Treatment characteristics were type of surgery and administration of chemotherapy. Tamoxifen use was not included because of the high rate of missing data for this variable. Cox proportional hazard regression models were used to estimate hazard ratios (HR) with 95% confidence intervals (CI). A multivariate Cox regression model was fitted that was based on all characteristics that had a *P*-value up to .10 in the univariate analysis. Variables determining molecular subtype were not included in multivariate analyses if molecular subtype was included. A 5% significance level was used and all tests are two-sided.

## Results

### Prognostic factors in young patients

A total of 9,938 early stage breast cancer patients participated in the four trials. Of this dataset, 1,192 (12%) patients were aged less than 40 years at the time of diagnosis. Paraffin embedded tumor material was obtained and processed into a tissue microarray for 549 patients aged less than 40 years (Table 1). Median age of these patients was 36.8 years. Tumors were subdivided according to molecular subtype: 111 patients (24%) aged less than 40 years had triple-negative tumors, 154 (34%) patients had luminal A tumors, 157 (34%) had luminal B tumors and 35 (8%) had HER2 tumors. Twenty-nine percent of tumors were p53 positive (Table 1). At time of analysis, 143 of 549 patients had died and 64 patients had developed distant metastases and were still alive.

**Table 1:** Characteristics of patients aged less than 40 years with immunohistochemistry results

Characteristic	All patients (*N *= 549)		Node negative patients (*N *= 341)	
	
	No. of Patients	%	No. of Patients	%
Age, years				
Median (range)	36.8 (23 to 40)		36.8 (25 to 40)	
Age distribution				
≥ 30 years	57	10	40	12
31 to 35 years	178	32	107	31
35 to 40 years	314	58	194	57
Pathological tumor size				
T1a and T1b	37	7	30	10
T1c	296	60	241	64
T2	158	32	82	26
T3	6	1	2	1
Missing	32		26	
Pathological nodal status				
Negative	341	63	341	100
Positive	204	37	0	0
Missing	4			
Surgery				
Breast conserving	446	81	299	88
Mastectomy	102	19	42	12
Missing	1		0	
Adjuvant chemotherapy^1^				
No	326	60	304	89
Yes	221	40	37	11
Missing	2		0	
ER status				
Positive	310	66	115	41
Negative	158	34	165	59
Missing	81		61	
PgR status				
Positive	223	48	141	51
Negative	241	52	136	49
Missing	85		64	
Tumor type				
Ductal	497	96	306	96
Lobular	17	3	10	3
Other	5	1	4	1
Missing	30		21	
Histological grade				
I	76	15	54	17
II	165	32	93	29
III	276	53	172	54
Missing	32		22	
Lymphangio invasion				
None	357	69	243	76
1-5 vessels	86	17	49	15
> 5 vessels	76	14	27	9
Missing	30		22	
HER2 status				
Negative	346	74	216	63
Positive	119	26	64	19
Missing	84		61	
P53 status				
Negative	331	71	198	72
Positive	133	29	78	28
Missing	85		65	
Molecular subtype				
Luminal A	154	34	79	29
Luminal B	157	34	86	32
HER-2	35	8	90	34
Basal (triple-negative)	111	24	14	5
Missing	92		72	

^1 ^EORTC trial 10854 randomized between one course of peri-operative chemotherapy which was not considered as prolonged chemotherapy.

Abbreviations: ER, estrogen receptor; HER2, human epidermal growth factor receptor 2; PgR, progesterone receptor.

At univariate analysis, pathological tumor size, nodal status, histological grade, lymphangio invasion, progesterone receptor status, molecular subtype, type of surgery and adjuvant chemotherapy were significantly associated with overall and distant metastasis free survival (Supplementary Table S1 in Additional file 1). HER2 and p53 status did not show an association with overall or distant metastasis free survival and were not included at subsequent multivariate analysis. At multivariate analysis (Table 2), pathologic tumor size, nodal status, and molecular subtype remained the only independent prognostic factors for both overall survival and distant metastasis free survival. For distant metastasis free survival, molecular subtype was a trend-significant prognostic factor (*P *= 0.06; Table 2).

**Table 2:** Multivariate analysis for prognostic factors in 549 patients aged less than 40 years

	Overall survival			Distant disease-free survival		
	**HR**	**95% CI**	***P***	**HR**	**95% CI**	***P***

pT2 + pT3	1.68	1.12 to 2.52	0.01	1.61	1.14 to 2.25	0.006
pN +	2.62	1.31 to 5.23	0.006	2.21	1.24 to 3.96	0.008
Lymphangio invasion			0.75			0.73
No vessels	1			1		
1 to 5 vessels	0.93	0.53 to 1.62		0.97	0.61 to 1.53	
> 5 vessels	1.18	0.71 to 1.97		1.17	0.75 to 1.83	
Molecular subtype			0.02			0.06
Basal	1			1		
Luminal A	0.50	0.29 to 0.86		0.69	0.44 to 1.08	
HER2	0.42	0.17 to 1.04		0.45	0.21 to 0.99	
Luminal B	0.92	0.56 to 1.48		1.01	0.67 to 1.53	
Breast conserving therapy	0.76	0.47 to 1.24	0.27	0.81	0.53 to 1.23	0.33
Adjuvant chemotherapy	0.68	0.35 to 1.33	0.26	0.65	0.37 to 1.13	0.13

Abbreviations: HR, hazard ratio; CI, confidence interval.

### Prognostic factors in young node negative patients

The subgroup of node negative patients aged less than 40 years consisted of 640 patients. Of 341 patients, tumor material was available for analysis (Table 1). Of these node negative patients, 304 (89%) patients did not receive adjuvant chemotherapy.

**Figure 1:**
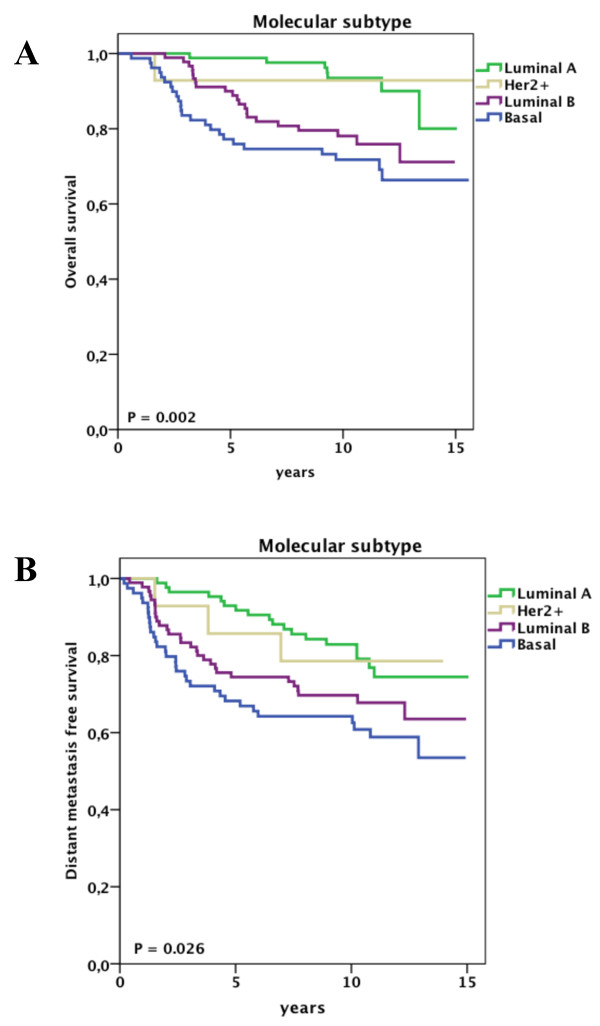
**Clinical outcome for node negative patients aged less than 40 years stratified by molecular subtype**. A, Overall survival. B, Distant disease-free survival.

**Figure 2:**
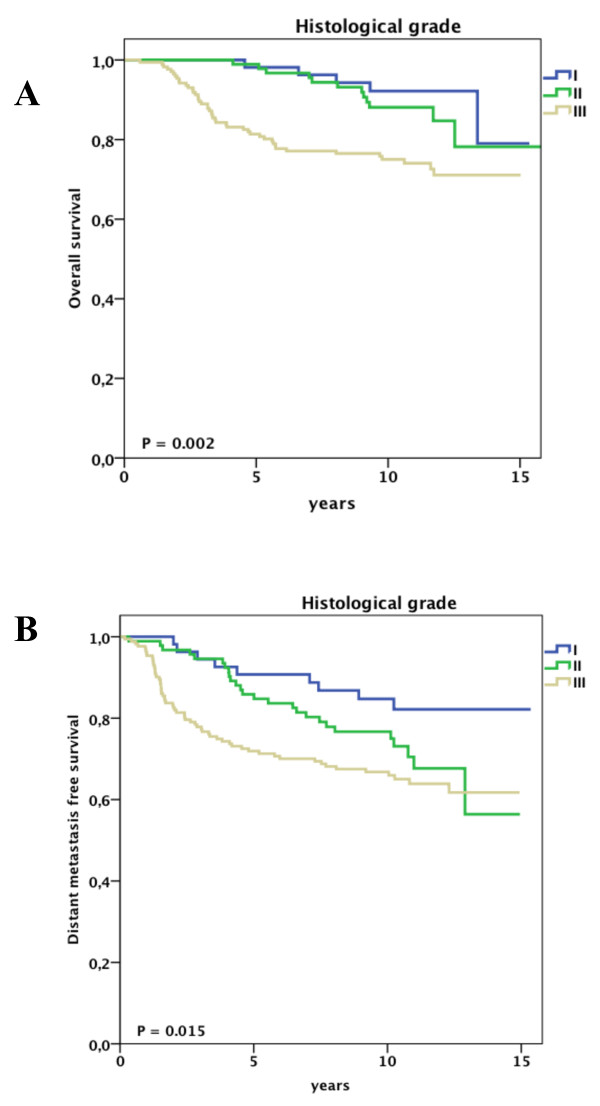
**Clinical outcome for node negative patients aged less than 40 years stratified by histological grade**. A, Overall survival. B, Distant disease-free survival.

**Figure 3:**
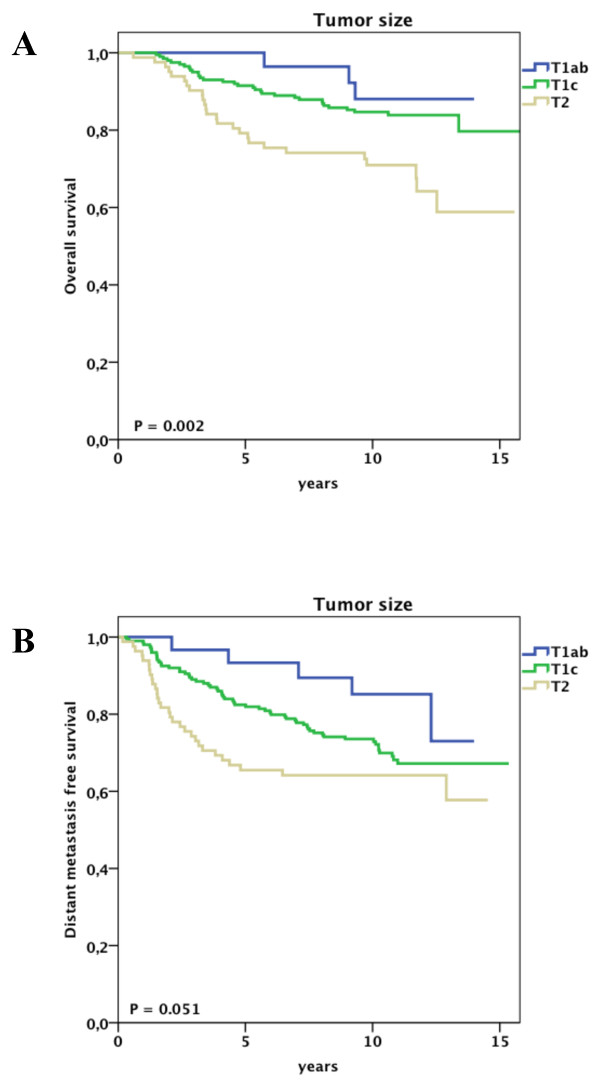
**Clinical outcome for node negative patients aged less than 40 years stratified by pathological tumor size**. A, Overall survival. B, Distant disease-free survival.

At univariate analysis, pathological tumor size, histological grade, estrogen receptor status and molecular subtype were significantly associated with overall survival and distant metastasis free survival (Supplementary Table S2 in Additional file 1). In addition, progesterone receptor status and the administration of adjuvant chemotherapy were significantly associated with overall survival. Figures 1, 2 and 3 show the impact of molecular subtype, histological grade and pathological tumor size, respectively, on overall and distant metastasis-free survival in the subgroup of node-negative patients aged less than 40 years. Ten-year survival rates for the molecular subtypes were 94% for luminal A, 93% for HER2, 78% for luminal B and 72% for basal (Figure 1A). Of note, the overall survival rate for young patients bearing a grade I tumor was 92% at 11 years of follow-up, whereas patients with grade III tumors had a survival rate of 72% (Figure 2A).

At multivariate analysis (Table 3), molecular subtype was associated with overall survival (*P *= 0.02; basal subtype versus luminal A subtype: hazard ratio (HR) 0.22, 95% CI 0.08 to 0.60, *P *= 0.003) and distant metastasis free survival (*P *= 0.08; basal subtype versus luminal A subtype: HR 0.46, 95% CI 0.25 to 0.85, *P *= 0.013).

**Table 3:** Multivariate analysis of prognostic factors in 341 node negative patients aged less than 40 years

	Overall survival			Distant disease-free survival		
	**HR**	**95% CI**	***P***	**HR**	**95% CI**	***P***

pT2 + pT3	1.75	0.99 to 3.10	0.06	1.33	0.83 to 2.15	0.24
Molecular subtype			0.02			0.08
Basal	1			1		
Luminal A	0.22	0.08 to 0.60		0.46	0.25 to 0.85	
HER2	0.25	0.03 to 1.85		0.53	0.16 to 1.73	
Luminal B	0.87	0.48 to 1.59		0.82	0.49 to 1.38	
Adjuvant chemotherapy	0.96	0.38 to 2.45	0.94			

## Discussion

In this study, we performed a retrospective pooled analysis to gain further insight into tumor characteristics of young breast cancer patients. In young patients, molecular subtype was the strongest prognostic factor of the covariates studied, distinguishing young patients with a favorable prognosis from young patients with an unfavorable prognosis.

In our study, young node negative patients with luminal A and HER2 tumors had excellent long-term overall survival rates of 94% and 93%, respectively, and distant disease-free survival rates of 83% and 79%, respectively, at 10-year follow-up (Figures 1A, B). Of note, only 3 out of 86 young node negative patients with luminal A tumors received adjuvant chemotherapy. Therefore, these data suggest that molecular subtype can be utilized in adjuvant treatment planning in young node negative patients. Recently, Cancello *et al*. showed that very young patients with basal, luminal B or HER2 breast cancer have a worse prognosis when compared with older patients with similar characteristics of disease [29].

Several authors have previously described the independent prognostic role of histological grade in young breast cancer patients. Sundquist *et al*. showed that patients bearing histological grade III tumors had a poor overall survival of 41% at the 11-year follow-up in a Swedish cohort of 107 patients aged less than 40 years [30]. Although histological grade was associated with overall survival, it did not reach statistical significance (Grade I versus III: HR 4.19; 95% CI 0.91 to 19.5), and nodal status was the strongest prognostic factor. Kollias *et al*. demonstrated in a cohort of 2,879 patients that the worse prognosis of the age group younger than 35 years (*N *= 120) was explained by the higher proportion of histological grade III breast cancers in the young group [31]. In a recent study, high histological grade and young age were identified as the most important risk factors for local relapse [32]. Our study suggests that histological grade (as part of molecular subtype) is an important prognostic factor in young breast cancer patients.

Among the established prognostic and predictive factors in young breast cancer patients, the estrogen receptor status is of particular interest. Recently, we showed that adjuvant chemotherapy provides limited survival benefit in hormone receptor positive young breast cancer patients [33]. The Korean Breast Cancer Society recently reported that young age was associated with a greater probability of death in breast cancer patients [34]. When studied in more detail, the survival difference was only found in the hormone receptor positive group [34]. As these data were collected from 1992 onwards, young hormone receptor positive patients received tamoxifen, in contrast to the patients in our study who were treated before adjuvant tamoxifen treatment became a standard in a large proportion of breast cancer patients with hormone receptor positive disease. The authors suggested that tamoxifen therapy might provide less survival benefit in young hormone receptor positive breast cancer patients as compared to older hormone receptor positive breast cancer patients. However, several trials have suggested an equal effect of endocrine therapy as compared to chemotherapy in hormone receptor positive premenopausal breast cancer patients [35,36]. In our data set of older EORTC trials, young hormone-receptor positive patients did not receive hormonal therapy. Notwithstanding, in our study, young patients with luminal A tumors had an excellent prognosis, which might have been augmented with the administration of hormonal therapy.

The current study has a number of limitations. First, the study design was a retrospective analysis of four heterogeneous randomized trials that were not primarily designed to test differences in outcome between young and old patients. Second, tumor material could not be collected for all patients aged less than 40 years and this could introduce selection bias. However, patient and traditional tumor characteristics were evenly distributed between the group for which tumor blocks were available and the group for which no tumor blocks could be collected (Supplementary Table S3 in Additional file 1). Third, information on tamoxifen use is largely missing. However, in the trials in which tamoxifen use was recorded, less than 5% of the patients younger than 40 years received tamoxifen. Despite these limitations, the current pooled analysis used individual patient data of four high-quality randomized controlled trials with renewed histopathological analysis to assess prognostic factors in young breast cancer patients. These data provide a robust estimate of breast cancer survival in relation to young age; and of the value of prognostic factors in young breast cancer patients. We believe our data justify a more critical view concerning current adjuvant chemotherapy guidelines in young low-risk breast cancer patients.

## Conclusions

The established prognostic factors molecular subtype, (including hormone receptor status, histological grade and HER2 receptor status), tumor size and nodal status remain independent prognostic factors on disease outcome in young breast cancer patients. In particular, molecular subtype was strongly associated with overall and distant metastasis free survival. Future treatment guidelines concerning young breast cancer patients should be refined based upon tumor characteristics, probably derived from microarray driven translational research projects, and not based upon age alone [37-39].

## Abbreviations

CMF: adjuvant chemotherapy with cyclophosphamide 100 mg/m^2 ^given orally on days 1 to 14, methotrexate 40 mg/m^2 ^given intravenously on Days 1 and 8, and 5-fluorouracil 600 mg/m^2 ^given intravenously on Days 1 and 8; EORTC: European Organisation for Research and Treatment of Cancer; FAC: doxorubicin 50 mg/m^2^, 5-fluorouracil 600 mg/m^2^, and cyclophosphamide 600 mg/m^2^; FEC: 5-fluorouracil 600 mg/m^2^, epirubicin 60 mg/m^2^, and cyclophosphamide 600 mg/m^2^; HR: hazard ratio

## Competing interests

The authors declare that they have no competing interests.

## Authors' contributions

JH participated in study design, carried out immunoassays and statistical analysis, and helped to draft the manuscript. SM performed statistical analysis, participated in data interpretation and drafted the manuscript. CV participated in study design and data interpretation. HP participated in design of the study and performed and coordinated statistical analysis. HB participated in study design and data interpretation. MV participated in design and coordination of the study, carried out immunoassays and helped to draft the manuscript. All authors read and approved the final manuscript.

## Supplementary Material

Additional file 1 **Supplementary tables S1-S3**. Supplementary table S1: Univariate regression analysis of clinicopathological characteristics for overall and distant disease-free survival of 549 patients aged less than 40 years. Supplementary table S2: Univariate regression analysis of clinicopathological characteristics for overall and distant disease-free survival of 341 node-negative patients aged less than 40 years. Supplementary table S3: Comparison of tumor size, lymph node status and administration of adjuvant chemotherapy between the group of patients aged < 40 years from whom tumor material was available for immunohistochemical analysis and the group of patients aged < 40 years from whom tumor material was not available. Click here for file
