# Supplementary material for: Impact of established prognostic factors and molecular subtype in very young breast cancer patients: pooled analysis of four EORTC randomized controlled trials
Source: Breast Cancer Res. 2011 Jun 24;13(3):R68. doi: 10.1186/bcr2908 (PMC3218957; doi:10.1186/bcr2908)
Supplement: Additional file 1 — Supplementary tables S1-S3. Supplementary table S1: Univariate regression analysis of clinicopathological characteristics for overall and distant disease-free survival of 549 patients aged less than 40 years. Supplementary table S2: Univariate regression analysis of clinicopathological characteristics for overall and distant disease-free survival of 341 node-negative patients aged less than 40 years. Supplementary table S3: Comparison of tumor size, lymph node status and administration of adjuvant chemotherapy between the group of patients aged < 40 years from whom tumor material was available for immunohistochemical analysis and the group of patients aged < 40 years from whom tumor material was not available. [file bcr2908-S1.DOC]

**Supplementary Table 1. Univariate analysis for prognostic factors in 549 patients aged less than 40 years**

|  | Overall survival | | | Distant disease-free survival | | |
| --- | --- | --- | --- | --- | --- | --- |
|  | HR | 95% CI | P | HR | 95% CI | P |
| pT2 + pT3 | 2.22 | 1.56-3.16 | <0.01 | 1.90 | 1.41-2.56 | <0.01 |
| pN + | 2.19 | 1.57-3.05 | <0.01 | 1.88 | 1.42-2.48 | <0.01 |
| Histological grade |  |  | <0.01 |  |  | <0.01 |
| I  II  III | 1  1.97  2.74 | 0.98-3.96  1.42-5.29 |  | 1  2.13  2.27 | 1.23-3.70  1.34-3.85 |  |
| Lymphangio invasion |  |  | 0.02 |  |  | 0.01 |
| No vessels  1-5 vessels  > 5 vessels | 1  0.96  1.80 | 0.58-1.57  1.18-2.74 |  | 1  0.97  1.71 | 0.64-1.47  1.18-2.47 |  |
| ER + | 0.77 | 0.53-1.12 | 0.17 | 0.92 | 0.67-1.26 | 0.61 |
| PgR + | 0.67 | 0.47-0.97 | 0.03 | 0.80 | 0.59-1.09 | 0.16 |
| HER2 + | 1.09 | 0.73-1.64 | 0.67 | 1.02 | 0.72-1.44 | 0.93 |
| Molecular subtype |  |  | 0.06 |  |  | 0.15 |
| Basal  Luminal A  Luminal B  HER2 | 1  0.56  0.96  0.61 | 0.34-0.92  0.62-1.50  0.27-1.37 |  | 1  0.75  1.07  0.60 | 0.50-1.14  0.73-1.58  0.29-1.24 |  |
| P53 + | 1.25 | 0.85-1.84 | 0.26 | 0.87 | 0.62-1.22 | 0.42 |
| Breast conserving therapy | 0.55 | 0.38-0.79 | <0.01 | 0.66 | 0.48-0.92 | 0.01 |
| Adjuvant chemotherapy | 1.87 | 1.34-2.61 | <0.01 | 1.55 | 1.17-2.05 | <0.01 |

Abbreviations: HR, hazard ratio; CI, confidence interval; ER, estrogen receptor; PgR, progesterone receptor; HER2, human epidermal growth factor receptor 2.

**Supplementary Table 2. Univariate analysis of prognostic factors in 341 node-negative patients aged less than 40** years.

|  | Overall survival | | | Distant disease-free survival | | |
| --- | --- | --- | --- | --- | --- | --- |
|  | HR | 95% CI | P | HR | 95% CI | P |
| pT2 + pT3 | 2.40 | 1.45-3.97 | <0.01 | 1.58 | 1.03-2.42 | 0.04 |
| Histological grade |  |  | <0.01 |  |  | 0.02 |
| I  II  III | 1  1.49  3.35 | 0.52-4.22  1.33-8.45 | 0.46  <0.01 | 1  1.86  2.60 | 0.90-3.96  1.29-5.23 | 0.11  <0.01 |
| Lymphangio invasion |  |  | 0.87 |  |  | 0.36 |
| No vessels  1-5 vessels  > 5 vessels | 1  1.14  1.21 | 0.57-2.25  0.52-2.83 |  | 1  1.24  1.55 | 0.72-2.14  0.80-3.01 |  |
| ER + | 0.57 | 0.34-0.97 | 0.04 | 0.63 | 0.41-0.96 | 0.03 |
| PgR + | 0.56 | 0.32-0.95 | 0.03 | 0.71 | 0.46-1.08 | 0.11 |
| HER2 + | 0.85 | 0.44-1.65 | 0.63 | 0.91 | 0.54-1.53 | 0.72 |
| Molecular subtype |  |  | <0.01 |  |  | 0.03 |
| Basal  Luminal A  Luminal B  HER2 | 1  0.23  0.72  0.22 | 0.10-0.54  0.40-1.30  0.03-1.59 |  | 1  0.43  0.75  0.47 | 0.24-0.76  0.45-1.24  0.14-1.52 |  |
| P53 + | 1.41 | 0.80-2.48 | 0.24 | 0.90 | 0.57-1.47 | 0.68 |
| Breast conserving therapy | 0.92 | 0.45-1.85 | 0.81 | 1.31 | 0.68-2.52 | 0.41 |
| Adjuvant chemotherapy ^2^ | 1.97 | 1.03-3.76 | 0.04 | 1.12 | 0.60-2.08 | 0.72 |

^1^ Two patients with pathological tumor size larger than 5 cm were excluded from the analysis. ^2^ 37 patients received adjuvant chemotherapy

Abbreviations: HR, hazard ratio; CI, confidence interval; ER, estrogen receptor; PgR, progesterone receptor; HER2, human epidermal growth factor receptor 2.

**Supplementary Table 3. Patient and tumor characteristics of patients aged < 40 years from whom tumor material was available for immunohistochemical analysis and of patients aged < 40 years from whom tumor material was not available.**

| Characteristic | Tumor material available | | Tumor material not available | | P |
| --- | --- | --- | --- | --- | --- |
|  | No. of Patients  (N = 549) | % | No. of Patients (N = 643) | % |  |
| Clinical tumor size  T1  T2  T3  Missing | 219  308  20  2 | 40  56  4 | 245  354  35  9 | 39  56  5 | 0.31 |
| Pathological nodal status  Negative  Positive  Missing | 341  204  4 | 63  37 | 392  236  15 | 62  38 | 0.96 |
| Adjuvant chemotherapy  No  Yes  Missing | 326  221  2 | 60  40 | 402  225  16 | 64  36 | 0.11 |
